# SARS-CoV-2 Serology Testing in an Asymptomatic, At-Risk Population: Methods, Results, Pitfalls

**DOI:** 10.3390/idr13040082

**Published:** 2021-10-21

**Authors:** Theodore Heyming, Kellie Bacon, Bryan Lara, Chloe Knudsen-Robbins, Aprille Tongol, Terence Sanger

**Affiliations:** 1 Children’s Hospital of Orange County, Orange, CA 92868, USA; theyming@choc.org (T.H.); kellie.bacon@choc.org (K.B.); bryan.lara@choc.org (B.L.); aprille.tongol@choc.org (A.T.); 2 Department of Emergency Medicine, University of California, Irvine, CA 92868, USA; 3 School of Medicine, University of Pittsburgh, Pittsburgh, PA 15213, USA; chloekr@g.ucla.edu; 4 Department of Electrical Engineering and Computer Science, University of California, Irvine, CA 92697, USA

**Keywords:** SARS-CoV-2, COVID-19, antibody testing, pediatric healthcare workers

## Abstract

The primary aim of this study was to determine the seroprevalence of SARS-CoV-2 antibodies in a population of pediatric healthcare workers (HCWs). This study was conducted 14 May–13 July 2020. Study participants included pediatric HCWs at a pediatric hospital with either direct patient contact or close proximity to patient-care areas. SARS-CoV-2 antibodies were assessed via the Wytcote Superbio SARS-CoV-2 IgM/IgG Antibody Fast Detection Kit and the Abbott Architect SARS-CoV-2 IgG assay. Participants underwent RT-PCR testing upon entry to the study and following rapid IgM+/IgG+ results; respiratory panel PCR (RP-PCR) was performed following IgM+ results. A total of 57 of 289 (19.7%) of participants demonstrated positive serology as assessed by the Wytcote rapid kit (12 on Day 1 and 45 throughout the study). However, only one of these participants demonstrated IgG+ serology via the Abbott assay. Two participants tested SARS-CoV-2+ via RT-PCR testing. One individual was adenovirus+ and enterovirus/rhinovirus+. In our study population, we observed a seroprevalence of SARS-CoV-2 antibodies of 0.35%. The lack of concordance between antibody tests suggests that the Wytcote rapid test kit may not be of use as a screening tool. However, the feasibility of the overall process indicates that a similar methodology may have potential for future epidemiologic surveillance.

## 1. Introduction

Since December 2019, more than 4 million people across the globe have died due to SARS-CoV-2 infection [1]. This highly transmissible virus and resultant pandemic necessitated the rapid development of tools to accurately detect rates of both current and historical infection [2]. Antibody titers have traditionally been employed as one measure for assessment of the immune response, and thus serologic testing has become a staple of pandemic modeling [3]. However, as with any screening method, especially in asymptomatic and low prevalence populations, it is important to select tests with an appropriate balance of sensitivity and specificity to reduce the incidence of false positives/negatives. The consequences of false positives/negatives must also be weighed together with the ease and accuracy of confirmatory testing.

Although the potential for increased exposure to SARS-CoV-2 among healthcare workers (HCWs) has led to numerous serologic studies, the pediatric HCW population remains somewhat less well surveyed [4,5,6,7,8,9]. Pediatric HCW are in general exposed to a large variety of infectious diseases, and during the COVID-19 pandemic, at increased risk for both known and unknown exposure to SARS-CoV-2 given the varied presentation of COVID-19 in children. The primary objective of this study was to determine the initial seroprevalence of SARS-CoV-2 antibodies in a population of asymptomatic pediatric HCWs. Secondary aims included assessing seroconversion and investigating the use of a rapid antibody test as a potential screening tool. Initial manufacturing data for the rapid antibody screening test used in this study included an estimated sensitivity and specificity for IgG of 100% and 85%, respectively, and an IgM sensitivity and specificity of 40% and 98%. The combined positive predictive values at a prevalence of 5% was reported to be 24.5%; the combined negative predictive value was estimated to be 100%, also at 5% prevalence.

## 2. Materials and Methods

This longitudinal descriptive study was conducted 14 May–13 July 2020 at a quaternary care children’s hospital. Study subjects were recruited via department-wide emails to the Emergency Department, Intensive Care Unit, interhospital transport team, and operative services teams, and included physicians, physician assistants, nurse practitioners, registered nurses, medical technicians, and secretaries with direct patient contact or who were in close proximity to patient-care areas. This study was approved by the institution’s institutional review board, and informed consent was obtained from all participants. Per this institution’s restrictions for on-campus work during the study period, all participants were afebrile and asymptomatic for each day of testing.

On Day 1 (D1), each subject underwent SARS-CoV-2 RT-PCR testing and two antibody detection tests, the Wytcote Superbio SARS-CoV-2 IgM/IgG Antibody Fast Detection Kit (Colloidal Gold) and the Abbott Architect SARS-CoV-2 IgG assay. The Wytcote rapid test assessed for IgM/IgG spike protein antibodies (results included individual IgM and IgG results); the Abbott Architect IgG assay assessed for IgG nucleocapsid antibodies. Wytcote testing was repeated weekly. Positive Wytcote results were repeated within 5 min; if the re-test was negative, this was considered a negative test (as positive tests precluded on-campus work, per agreement with hospital administration, these were repeated to reduce the likelihood of false positives). Any new IgM+ results via the rapid Wytcote test were followed by repeat RT-PCR and Abbot testing along with an initial BioFire FilmArray Respiratory Panel PCR (RP-PCR). Wytcote rapid IgG+ results were followed by RT-PCR and Abbott testing. Any participant who became symptomatic during the study period underwent Wytcote, Abbott, and RT-PCR testing. All specimen collection was performed per manufacturer/CDC specifications. Test-specific data may be found in the Appendix A.

## 3. Results

A total of 289 subjects enrolled in this study; two withdrew prior to antibody testing, and an additional five withdrew at various stages, including one participant who tested SARS-CoV-2 positive via RT-PCR on D1. Participant demographics are displayed in Table 1.

On D1, 12 participants tested positive via Wytcote; 2 were IgM+, 6 were IgG+; and 4 were IgM+/IgG+. Only 1 of these 12 participants ever demonstrated IgG+ serology via the Abbott assay, and that Abbott assay result was from D1. RT-PCR performed the same day for SARS-CoV-2 was also positive. This participant did not complete any additional testing. No other participant was SARS-CoV-2 positive on RT-PCR testing on D1. In the 5 days prior to D1, 44.6% of participants reported directly caring for SARS-CoV-2 positive patients (while wearing appropriate PPE). No participant reported testing SARS-CoV-2 + at any point prior to D1.

**Figure 1:**
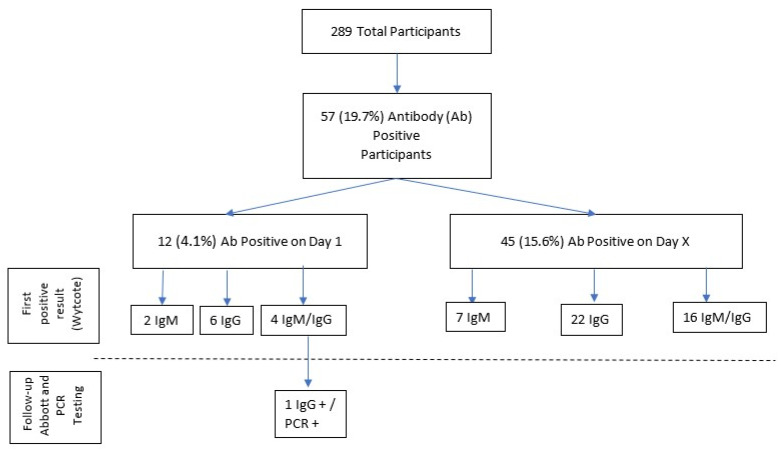
Flow Diagram of Results for Antibody Positive Participants. Positive antibody results from participants over time, broken into first positive Wytcote result, IgG follow-up testing via the Abbott assay and PCR follow-up testing. IgM—Immunoglobulin M, IgG—Immunoglobulin G, Ab-Antibody.

Throughout the study, 45 additional participants received positive Wytcote results (Figure 1). On the respective days that these participants first demonstrated positive results, 7 were IgM+, 22 were IgG+, and 16 were IgM+/IgG+. Follow-up Wytcote testing on these participants was intermittently negative for some individuals. None of these 45 participants ever demonstrated IgG+ serology via the Abbott assay. Only one participant tested SARS-CoV-2 positive via RT-PCR after D1; all other follow up RT-PCR testing was negative. In the case of the single participant who tested SARS-CoV-2 positive via RT-PCR after D1, both Wytcote and Abbott antibody tests were negative for this individual on the day that the RT-PCR was positive (given the negative antibody results, this PCR result is not included in Figure 1). This participant became symptomatic during the study period, and no further testing was obtained.

The test–retest reliability results of the Wytcote kit were 46.8% for IgM+ and 51.6% for IgG+ (111/117 initial IgM+ tests were retested and 159/161 initial IgG+ tests were retested).

A total of 38 RP-PCR tests were run on samples from 30 participants; none were positive for the four tested coronaviruses; one specimen was adenovirus/enterovirus/rhinovirus positive.

## 4. Discussion

In this study, the observed seroprevalence of COVID-19 IgG antibodies, as documented by the Abbott assay, was 0.35%. This is lower than the observed seroprevalence of 1.06% reported by Brant-Zawadzki et al. in a study of HCWs at a regional hospital system in the same county and time period as the current study [10]. Community prevalence in the county at the time was estimated to be 4.4% [11]. The positivity rate (per RT-PCR) for patients tested at this institution during the study period was 8.1%. There have been several studies examining the prevalence of SARS-CoV-2 and the seroprevalence of IgM/IgG antibodies in HCWs compared to community levels, and results are varied [4,12,13,14,15,16,17,18]. Though there have been fewer studies of pediatric HCWs to date, rates of positive serology in pediatric HCWs have consistently been found to be lower or equal to those measured in the general population, with the reported range of positive serology ranging from 0–16.9% [5,6,7,8,9,19,20,21].

In our study, the consistency of the reported findings was complicated by the considerable discrepancy between the Wytcote test results and the Abbott assay. Only 1 out of 82 IgG+ Wytcote tests (1.2%) was confirmed positive via the Abbott assay. The low test–retest reliability and poorer overall specifications of this Wytcote product, in combination with its voluntary removal from the market, suggest that the results from the Abbott assay are likely more accurate. In addition, the sensitivity and specificity of the Abbott test have been further validated [22,23]. In light of this, it is difficult to confidently interpret the IgM findings obtained with the Wytcote product. It is of note that only one of the IgM+ participants received a positive RT-PCR for SARS-CoV-2 (on D1), and RP-PCR testing was negative for other coronaviruses. No IgM+ individuals who initially tested positive only for IgM ever developed documented IgG antibodies (per the Wytcote test) during the course of the study, potentially suggesting that the IgM results that were obtained were false positives [24,25]. It is possible that IgG antibodies developed but remained at undetectable levels or that they developed outside of the study period. However, the overall pattern of the results obtained here suggests that there was no observed seroconversion throughout the course of the study.

In a pandemic, it is challenging yet essential to accurately gauge community disease burden in order to design effective public health policies. Antibody serology testing can be especially useful in a setting where there is insufficient surveillance testing and the presence of asymptomatic cases [26,27,28,29]. In addition, previous studies have demonstrated diverse antibody responses to COVID-19, with less clinically severe infections generally corresponding to weaker antibody response as well as variable rates of antibody level decay [3,30,31,32,33]. It is not yet understood how this may pertain to future immunity [31,34]. As efforts to understand the spread of COVID-19 continue, there appears to be a clear role for sero-epidemiology. Pollán et al. demonstrated the successful use of a point-of-care test, with reliability similar to that of the Abbott assay, in a study of over 60,000 Spanish residents [28]. Additionally, these concepts are of importance in children; as they are still more likely to be asymptomatic carriers, case counts in children have risen compared to at the start of the pandemic, and children have largely returned to in-person schooling.Interestingly [35], the Infectious Diseases Society of America guidelines, published in August 2020, after completion of the present study, recommend against SARS-CoV-2 IgG/IgM combination testing to assess for previous infection and do not make recommendations for or against the use of IgM testing for the same purposes; however, they commented that these guidelines were based on a “very low certainty of evidence”. Importantly, they note that to be of use, serology assays must have excellent specificity and sensitivity, especially in an asymptomatic/low prevalence population; unfortunately, the Wytcote test used in this study did not seem to demonstrate the necessary sensitivity and specificity [36].

## 5. Limitations of the Study

There are several limitations to this study in addition to the previously discussed lack of concordance between antibody tests. Employees at this institution were not permitted to work on campus if febrile or symptomatic, and this may have contributed to low observed prevalence, although seven subjects did not complete the study. False negatives may have also contributed to the relatively low observed seroprevalence, especially considering that the only negative results verified as such were those obtained on D1 [2].

## 6. Conclusions

In our study population, the observed seroprevalence of COVID-19 IgG antibodies as measured by the Abbott assay was 0.35%. The extremely low concordance between the Wytcote rapid test kit and the Abbott assay suggests that the Wytcote Superbio rapid test kit may not be of use as a screening tool. However, the techniques described may be used in the development of future large-scale 2-step antibody screening processes.

## Figures and Tables

**Table 1:** Descriptive Statistics (*N* = 289).

▪ Age	18–30	27.68%
	31–40	32.87%
	41–50	24.57%
	Over 50	14.88%
▪ Sex	Male	25.61%
	Female	74.39%
▪ Race/Ethnicity	American Indian or Alaska Native	0.35%
	Asian or Asian American	15.57%
	Black or African American	1.38%
	Hispanic/Latino	19.38%
	Multiracial	5.19%
	Other	2.77%
	Pacific Islander	1.38%
	White or Caucasian	53.98%
▪ Years of Experience	Range	1–52 years
	Mean	13.21
▪ Position Type	Full Time	74.39%
	Part Time	22.15%
	Per Diem	3.46%
▪ Position Title	Physician	20.07%
	PA/NP ^1^	2.77%
	Registered Nurse	51.21%
	Technician	15.22%
	Monitor Tech/Unit Secretary/Administration/Other	10.73%

^1^ PA-Physician Assistant, NP-Nurse Practitioner.

## Data Availability

Deidentified individual participant data will not be made available.

## Appendix A

The Wytcote Superbio SARS-CoV-2 IgM/IgG Antibody Fast Detection Kit (Colloidal Gold) provides a qualitative assessment of IgM and IgG antibodies to the spike protein of SARS-CoV-2 from a blood sample obtained via finger prick in approximately 8 min. According to the initial evaluation by the NCI through the FDA’s performance validation study effort, IgM sensitivity was 40%, and the specificity was 98.8%. IgG sensitivity was 100%, and the specificity was 85.0%, for a combined sensitivity and specificity of 100% and 83.8%, respectively. At a prevalence of 5%, the PPV was 24.5%, and the NPV was 100%. (https://www.accessdata.fda.gov/cdrh_docs/presentations/maf/maf3318-a001.pdf, accessed on 10 January 2021) The Wytcote kit was not validated by this institution’s lab prior to use.

This detection kit was granted an EUA from the FDA in the first months of the COVID-19 pandemic, and since the termination of this study, it has been voluntarily withdrawn from the market.

The Abbott Architect SARS-CoV-2 IgG assay demonstrated an IgG sensitivity of 90.0% and a specificity of 100%, with a PPV of 100% and a NPV of 99.5% at a 5% prevalence on evaluation by the CDC (https://www.accessdata.fda.gov/cdrh_docs/presentations/maf/maf3305-a001.pdf, accessed on 10 January 2021)). Bryan et al. evaluated the Abbott antibody assay and found a sensitivity of 100% (13 days post-positive PCR/17 days post-symptom onset) and a specificity of 99.9%. The 7 day post-symptom onset corresponded to a sensitivity of 53.1%; 10 day onset corresponded to to 82.4%; and 14 day corresponded to 96.9%. The Abbott assay remains approved under EUA by the FDA. This assay was validated by the study institution’s lab prior to use.

BioReference Laboratories’ validated NPS SARS-CoV-2 RT-PCR assay was used to detect current viral load.

The BioFire FilmArray Respiratory Panel 2—IVD assessed for the following viruses and bacteria: Adenovirus, Coronavirus 229E, Coronavirus HKU1, Coronavirus NL63, Coronavirus OC43, Human Metapneumovirus, Human Rhinovirus/Enterovirus, Influenza A, Influenza B, Parainfluenza Virus 1, Parainfluenza Virus 2, Parainfluenza Virus 3, Parainfluenza Virus 4, Respiratory Syncytial Virus, Bortadella parapertussis, Bortadella pertussis, Chlamydia pneumoniae, Mycoplasma pneumoniae.
